# Longitudinal assessment of utilities in patients with migraine: an analysis of erenumab randomized controlled trials

**DOI:** 10.1186/s12955-019-1242-6

**Published:** 2019-11-12

**Authors:** Gian Luca Di Tanna, Joshua K. Porter, Richard B. Lipton, Anthony J. Hatswell, Sandhya Sapra, Guillermo Villa

**Affiliations:** 1 Economic Modeling Center of Excellence, Amgen (Europe) GmbH, Rotkreuz, Switzerland; 2 The George Institute for Global Health, Newtown, New South Wales Australia; 3 Albert Einstein College of Medicine, Bronx, New York, NY USA; 4 Delta Hat Limited, Nottingham, UK; 5 Amgen Inc., Thousand Oaks, CA USA

**Keywords:** Utility, Migraine, Longitudinal, Modelling

## Abstract

**Background:**

Cost-effectiveness analyses in patients with migraine require estimates of patients’ utility values and how these relate to monthly migraine days (MMDs). This analysis examined four different modelling approaches to assess utility values as a function of MMDs.

**Methods:**

Disease-specific patient-reported outcomes from three erenumab clinical studies (two in episodic migraine [NCT02456740 and NCT02483585] and one in chronic migraine [NCT02066415]) were mapped to the 5-dimension EuroQol questionnaire (EQ-5D) as a function of the Migraine-Specific Quality of Life Questionnaire (MSQ) and the Headache Impact Test (HIT-6™) using published algorithms. The mapped utility values were used to estimate generic, preference-based utility values suitable for use in economic models. Four models were assessed to explain utility values as a function of MMDs: a linear mixed effects model with restricted maximum likelihood (REML), a fractional response model with logit link, a fractional response model with probit link and a beta regression model.

**Results:**

All models tested showed very similar fittings. Root mean squared errors were similar in the four models assessed (0.115, 0.114, 0.114 and 0.114, for the linear mixed effect model with REML, fractional response model with logit link, fractional response model with probit link and beta regression model respectively), when mapped from MSQ. Mean absolute errors for the four models tested were also similar when mapped from MSQ (0.085, 0.086, 0.085 and 0.085) and HIT-6 and (0.087, 0.088, 0.088 and 0.089) for the linear mixed effect model with REML, fractional response model with logit link, fractional response model with probit link and beta regression model, respectively.

**Conclusions:**

This analysis describes the assessment of longitudinal approaches in modelling utility values and the four models proposed fitted the observed data well. Mapped utility values for patients treated with erenumab were generally higher than those for individuals treated with placebo with equivalent number of MMDs. Linking patient utility values to MMDs allows utility estimates for different levels of MMD to be predicted, for use in economic evaluations of preventive therapies.

**Trial registration:**

ClinicalTrials.gov numbers of the trials used in this study**:** STRIVE, NCT02456740 (registered May 14, 2015), ARISE, NCT02483585 (registered June 12, 2015) and NCT02066415 (registered Feb 17, 2014).

## Background

Cost-effectiveness analyses are often used by reimbursement agencies to make decisions on whether to reimburse new healthcare interventions. Health-related quality of life (HRQoL) values can be expressed as utility scores, which capture social preferences for different health states [1]. Often, studies with HRQoL outcomes have repeated assessment over time and are longitudinal in nature. Analysing longitudinal data can present various challenges, such as missing data or the variations in patient HRQoL over time [2]. Therefore, it is important to consider the appropriate model when analysing longitudinal HRQoL data.

Regression models have been used to estimate treatment-effect impact on HRQoL [3]. Simple linear regression models, however, may not be optimal because health utility measures, including measures of HRQoL, may be multimodal and have ceiling effects, floor effects or skewed distributions [3–5]. In these circumstances, multivariable analyses, such as linear mixed models, may be more appropriate to estimate the changes in HRQoL over time. Linear mixed models, which are an extension of simple linear models and contain both fixed and random effects, can overcome the limitations associated with a longitudinal data set [2]. Recent analyses have demonstrated the suitability of the use of linear mixed models to measure HRQoL in longitudinal cohorts in a range of disease areas [2, 6]. Anink et al. applied linear mixed models to examine HRQoL data in patients with juvenile idiopathic arthritis [6]. Wailoo et al. demonstrated the use of bespoke mixed models to model the 5-dimension EuroQol questionnaire (EQ-5D) in patients with ankylosing spondylitis [4]. Griffiths et al. estimated utility values from mixed regression models using EQ-5D data in patients with chronic heart failure [2].

According to the National Institute for Health and Care Excellence ‘Guide to the Methods of Technology Appraisal’, EQ-5D is the preferred method for measuring utilities [7, 8]. Utilities can be estimated from individual patient-level data collected as part of clinical studies (or extrapolated in the absence of long-term data) [9]. However, the collection of EQ-5D utilities is not always appropriate or possible in every disease state, so other methods may be used [7, 8]. Limited guidance exists on approaches to extrapolating outcomes such as utilities [10]. Applying existing algorithms is one of the options to derive utilities for health-state estimates when they are not available from the original data set [10, 11]. Extrapolation methods should, however, consider processes that influence utilities that may not be linked to clinical events (e.g. past medical history of a patient or changes in clinical practice over time that may affect current practice) [10]. These considerations are particularly relevant to migraine, a chronic neurological disorder with episodic attacks of headache and an array of other symptoms [12]. Migraine is a debilitating disease in which utilities are typically measured via the Health Utilities Index (HUI) or the EQ-5D [13–16]. Migraine has considerable negative effects on a person’s HRQoL, in addition to a high economic burden due to high direct costs (physician visits, emergency department visits, etc.) and indirect costs (lost work days, decreased productivity at work, etc.) [17]. Migraine can be divided into two categories based on the number of days on which patients have a headache in a 28-day month. Chronic migraine (CM) is defined as experiencing ≥15 monthly headache days (MHD) for ≥3 or more months, 8 of which meet the criteria for migraine and/or respond to migraine-specific treatments [12]. Episodic migraine (EM) is defined as experiencing ≤14 MHD [18–20].

Reduction in the frequency of monthly migraine days (MMD) is an important measure in the efficacy of migraine prophylaxis; however, there are limited data on the relationship between migraine frequency and health status [15]. Furthermore, patient-level data collected within the time frame of a clinical study often cover too short a duration to assess the likely costs and benefits that may yield over an individual’s entire lifetime [10]. Preventive treatment can reduce the burden and disability associated with migraine [21]. Erenumab is a fully human monoclonal antibody that specifically blocks the calcitonin gene-related peptide receptor complex [22] and has been shown to have a favourable safety and efficacy profile in phase 2 and phase 3 clinical studies [23–25]. In 2018, erenumab was approved by the US Food and Drug Administration for the prevention of migraine in adults [26].

The pivotal erenumab clinical studies included endpoints that recorded HRQoL data. This study aimed to leverage the HRQoL data from these studies to estimate patient utility values associated with specific levels of MMD. Various models for utilities in the longitudinal framework were compared using the observed utility data. Quantifying how the primary outcomes of the clinical studies, that is, MMDs, relate to utility values is important to inform cost-effectiveness analyses of preventive therapies such as erenumab [27].

## Methods

### Data source

The populations assessed in the models are the populations of three pivotal erenumab clinical studies [23, 24, 28]. In the phase 3 (NCT02456740) STRIVE (Study to Evaluate the Efficacy and Safety of Erenumab in Migraine Prevention), 955 patients with EM were enrolled. In the phase 3 (NCT02483585) ARISE (A phase 3, Randomized, double-blind, placebo-controlled Study to Evaluate the efficacy and safety of AMG 334 in migraine prevention), 577 patients with EM were enrolled and in the phase 2 study, 667 patients with CM were enrolled. The EM studies recruited individuals with ≤14 MHD and 4–14 MMDs per 28 days and the CM study recruited individuals with ≥15 HDs per 28 days and > 8 MD. To generalize the influence that MMD frequency has on patient utility values, the patient-reported outcomes for the placebo and erenumab (70 mg and 140 mg) arms of the three studies were combined to produce a complete migraine data set. Patient-level data were obtained for the participants in each study, with the following variables extracted for use in the analysis as the covariate set: participant identification (ID), study ID, age (continuous), sex (categorical), race (categorical), MMD at baseline (count), MMD (count) and treatment status (categorical). Covariates were selected based on known associations and clinical advice from experts in the field [29, 30]. Study-level effects were originally included in the hierarchical models, but as they demonstrated a negligible amount of variability between the studies, this layer was removed. As the objective of the analysis was to estimate patient utility based on MMD across the full migraine spectrum, combined models based on both EM and CM were fitted. Furthermore, the trials were comparable in terms of patients characteristics [31], therefore only patients level data were retained in the multilevel models presented here.

### Data description

Patient utilities in the model were estimated as a function of MMD. For this analysis, MMD refers to the number of migraine days during a 28-day period. In the three studies, patients’ HRQoL and daily functioning were collected in a monthly assessment, using the Headache Impact Test (HIT-6™) [32] and the Migraine-Specific Quality of Life Questionnaire (MSQ) [33]. The HIT-6 is designed to provide a global measure of adverse headache impact. Via a HRQoL questionnaire, the HIT-6 evaluates six content areas: pain, role functioning, social functioning, energy/fatigue, cognition and emotional distress [34]. The MSQ is a 14-item HRQoL questionnaire that measures three dimensions of functional status (role prevention, role restrictive and emotional function) specific to migraine [33, 34]. Both the MSQ and the HIT-6 have been shown to be valid and reliable tools for measuring the adverse impact of headache [32, 35]. Disease-specific patient-reported outcomes from the studies were mapped to the EQ-5D using published algorithms. The mapping algorithms applied here have been previously published by Gillard et al., and these algorithms have been validated to support the analysis of onabotulinum toxin A (Botox®) trial data (see Online Resource: Additional file 2: Table S1) [34]. The size of the prediction error of the validated models was assessed using root mean squared error (RMSE) and mean absolute error (MAE).

In addition to the complete case analysis, a multivariate imputation by chained equations (MICE; fully conditional specification [FCS] algorithm) was performed with the assumption that data were missing at random. The MICE-FCS technique is a standard methodology for dealing with missing data and is also appropriate in the context of longitudinal data. The variables used in the imputation model were mapped MSQ, mapped HIT-6, treatment, baseline MMD, MMD, visit, age, sex and race. This imputation assessed the robustness of the results according to the presence of missing data and was constructed on a FCS [36] and based on 15 multiple imputed data sets [37].

### Utility regression models

For this analysis, four models were assessed: (1) a linear mixed effects model with REML, (2) a fractional response model with logit link, (3) a fractional response model with probit link and (4) a beta regression model. Multilevel modelling approaches were chosen in order to take account of the longitudinal framework of the three trials, which included measurements collected from the same participants at repeated intervals over the course of the studies. These multilevel modelling approaches were used to enable the clustering of observations at the patient level.

In all models, the covariate set was examined. The mean predicted utilities by MMD (and by treatment status) were estimated with standard errors using the delta method [38]. Multilevel modelling techniques estimate the differences between individuals, acknowledging that measurements from the same person over time are much more likely to be correlated than measurements from different individuals [39].

In all four models the covariates included were as follows: treatment status (erenumab 70 mg or 140 mg vs placebo), age, sex (female vs male), race (black, Asian, other vs white), MMD at baseline, MMD at each visit and visit. The mean predicted utilities by MMD (and by treatment status) were estimated with standard errors using the delta method [38].

#### Linear mixed effects model with REML

A linear mixed effects model has been estimated with the REML method as a random-effects at the patient level, to estimate subject-specific effects and to provide distilled estimates of the specified covariates (the fixed component of the model) and estimates of the random variation according to the individuals [2, 40]. Acknowledging that a standard linear regression model (although hierarchical) is not well suited for an outcome that has a delimited unit interval such as utility values, which are typically characterized by a truncated support at both ends of the distribution (usually ranging between 0 and 1) and with heteroscedasticity (i.e. the variance of the residuals is not constant) as an integral part of such limited dependent variables [41], models fitted under the generalized linear model (GLM) framework have been shown to produce better estimates than those estimated by the linear model [42].

#### Linear mixed effects model with REML

$$ E\left({U}_{ti}\mid X\right)={\beta}_0+\overline{\beta_1}{Treatment}^J+{\beta}_2{BaselineMMD}_i+{\beta}_3{MMD}_{ti}+{\beta}_4{Age}_i+{\beta}_5{Female}_i+\overline{\beta_6}{Race}^K+\overline{\beta_7}{Visit}^T+{u}_i $$

Where *t* = 0, 4, 8, 12, 16, 20, 24^a^ weeks and *i* = 1, 2,.., 2197 patients; *J* is erenumab 70 mg, erenumab 140 mg (vs placebo), *K* is black, other (vs white).

^a^16–24 weeks for EM studies only.

#### Fractional response models with a logit link function or a probit link function

Another valid strategy for handling proportions data in which zeros and ones may appear (as well as intermediate values) [43] is the fractional response model [44]. This model can be estimated via the GLM suite using the logit link function (i.e. the logit transformation of the response variable) or the probit link function [45]. Robust standard errors have been estimated allowing for clustering at individual participant level.

#### Fractional response models with a logit link function or a probit link function

$$ E\left({U}_{ti}|\ X\right)=G\left(\overline{\beta_1}{Treatment}^J+{\beta}_2{BaselineMMD}_i+{\beta}_3{MMD}_{ti}+{\beta}_4{Age}_i+{\beta}_5{Female}_i+\overline{\beta_6}{Race}^K+\overline{\beta_7}{Visit}^T\right) $$

where *G*(.) is a probit or logit function.

#### Beta regression model

The fourth model fitted is a beta regression that is useful to model continuous, 0–1 bounded and beta distributed outcomes. In the data set for this analysis, outcomes were constrained to have values higher than 0 and less than 1. Because some patients had a mapped utility (EQ-5D) value of 1, these values were decreased by 1.110^e-16^, a marginal decrease to ensure minimal difference from the original values. As for the fractional response models, robust standard errors were estimated.

The density of the beta-distributed dependent variable U conditional on covariates X can be written as

$$ f\left(U;{\mu}_X,{\varphi}_X\right)=\frac{\Gamma \left({\varphi}_X\right)}{\Gamma \left({\mu}_X{\varphi}_X\right)\Gamma \left[\left(1-{\mu}_X\right){\varphi}_X\right]}{U}^{\mu_X{\varphi}_X-1}{\left(1-U\right)}^{\left(1-{\mu}_X\right){\varphi}_X-1} $$

Where *μ*_*X*_ = *E*(*U*_*ti*_| *X*) is linked to the covariates set by *g*(*μ*_*X*_) (a logit function of the linear predictor described above) and *φ*_*X*_ is the scale parameter of the conditional variance of U.

Goodness of fit of the regression models was assessed by RMSE, MAE and visual assessments.

All statistical analyses have been conducted using Stata 15 (StataCorp 2017 Stata Statistical Software, Release 15; StataCorp LLC, College Station, TX, USA).

## Results

### Baseline characteristics

The analysis sample included data from 2199 patients. Characteristics of the patients from the three studies are presented in Table 1. Baseline characteristics were similar across the three studies. For example, the average age was in the range 40.4–42.9 years across the three studies. The majority of patients in all studies were white and female, as is typical in migraine.

**Table 1:** Baseline characteristics of patients in the erenumab clinical trials [23, 24, 28]

Characteristic	Episodic migraine (NCT02456740) STRIVE			Episodic migraine (NCT02483585) ARISE		Chronic migraine (NCT02066415)		
	Placebo	Erenumab 70 mg	Erenumab 140 mg	Placebo	Erenumab 70 mg	Placebo	Erenumab 70 mg	Erenumab 140 mg
Number of patients	319	317	319	291	286	286	191	190
Mean age, years (SD)	41.3 (11.2)	41.1 (11.3)	40.4 (11.1)	42.2 (11.5)	42.3 (11.4)	42.1 (11.3)	41.4 (11.3)	42.9 (11.1)
Sex, n (%)								
Male	45 (14.1)	48 (15.2)	47 (14.7)	44 (15.1)	41 (14.3)	60 (21.0)	25 (13.1)	30 (15.8)
Female	274 (85.9)	269 (84.8)	272 (85.3)	247 (84.9)	245 (85.7)	226 (79.0)	166 (86.9)	160 (84.2)
Race, n (%)								
White	276 (86.8)	280 (88.6)	293 (91.9)	259 (89.0)	259 (90.6)	268 (93.7)	176 (92.1)	184 (96.8)
Black	24 (7.5)	24 (7.6)	18 (5.6)	27 (9.3)	24 (8.4)	11 (3.8)	10 (5.2)	6 (3.2)
Other	18 (5.7)	12 (3.8)	8 (2.5)	5 (1.7)	3 (1.0)	7 (2.5)	5 (2.6)	0 (0.0)
MMD	8.2 ± 2.5	8.3 ± 2.5	8.3 ± 2.5	8.4 ± 2.6	8.1 ± 2.7	18.2 ± 4.7	17.9 ± 4.4	17.8 ± 4.7

*Abbreviations: ARISE* A phase 3, Randomised, double-blind, placebo-controlled Study to Evaluate the efficacy and safety of AMG 334 in migraine prevention, *MMD* monthly migraine day, *SD* standard deviation, *STRIVE* Study to evaluate the efficacy and safety of erenumab in migraine prevention

### Validated mapping algorithms

In episodic migraine, the HIT-6 and MSQ algorithms explained 8 and 14% of the variance, respectively, as measured by adjusted R^2^, and had similar prediction errors (RMSE of 0.32). In chronic migraine, the HIT-6 and MSQ algorithms explained 19 and 30% of the variance, respectively, and had similar prediction errors (RMSE of 0.33 and 0.32).

### Comparison of regression outputs and utility values

Four regression models were fitted using mapped utility values, MMD and treatment group (erenumab 70 mg, 140 mg and placebo), adjusting for age, sex, race and baseline MMD in the various time periods considered. Mapped utility values are described in Table 2 and the distribution of the mapped utilities values are shown in Additional file 1: Figure S1 HIT-6 mapped mean (standard deviation [SD]) utility values were consistently higher than MSQ mapped mean (SD) utility values for patients treated with erenumab 70 mg and 140 mg, and for patients receiving placebo. HIT-6 and MSQ mapped mean (SD) utility values increased over time up to week 16 for patients treated with erenumab 70 mg and 140 mg, and for patients treated with placebo. MSQ and HIT-6 mapped mean utility values were lowest in the 4 weeks before randomization to treatment or placebo (week 0). Patients at week 0 were predicted to have MSQ mapped mean (SD) utility values of 0.633 (0.163) and 0.617 (0.176) for patients treated with erenumab 70 mg and 140 mg, respectively, and 0.615 (0.173) for patients receiving placebo (Table 2).

**Table 2:** Mean utility (EQ-5D) values extrapolated from MSQ and HIT-6

Week, mean (SD)	Placebo		Erenumab 70 mg		Erenumab 140 mg	
	MSQ	HIT-6	MSQ	HIT-6	MSQ	HIT-6
0	0.615 (0.173)	0.671 (0.136)	0.633 (0.163)	0.677 (0.125)	0.617 (0.176)	0.675 (0.145)
4	0.675 (0.161)	0.721 (0.135)	0.716 (0.142)	0.747 (0.125)	0.716 (0.154)	0.761 (0.147)
8	0.687 (0.160)	0.732 (0.137)	0.726 (0.141)	0.771 (0.133)	0.729 (0.153)	0.775 (0.147)
12	0.687 (0.160)	0.734 (0.139)	0.730 (0.146)	0.776 (0.134)	0.737 (0.144)	0.787 (0.145)
16	0.736 (0.120)	0.781 (0.119)	0.765 (0.111)	0.810 (0.122)	0.775 (0.094)	0.828 (0.114)
20	0.731 (0.127)	0.786 (0.128)	0.771 (0.100)	0.813 (0.121)	0.776 (0.093)	0.824 (0.114)
24	0.729 (0.129)	0.781 (0.132)	0.763 (0.111)	0.812 (0.122)	0.776 (0.091)	0.824 (0.114)

*Abbreviations: EQ-5D* 5-dimension EuroQol questionnaire, *HIT-6* Headache Impact Test, *MSQ* Migraine-Specific Quality of Life Questionnaire, *SD* standard deviation

**Fig. 1:**
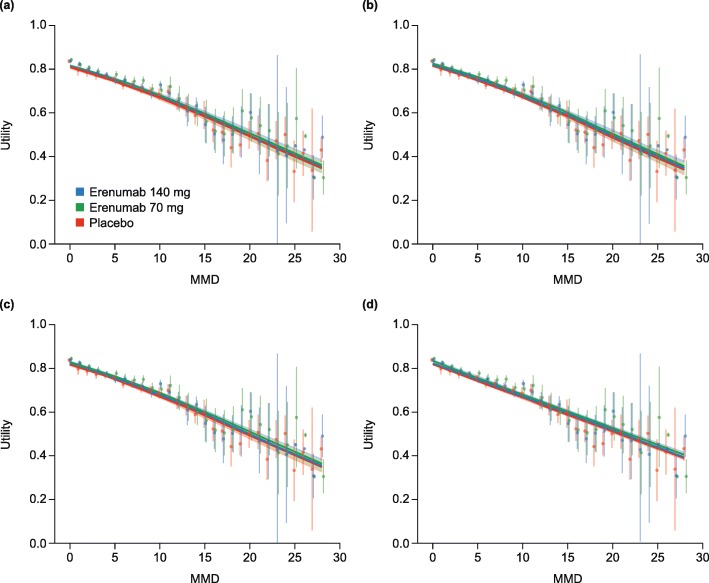
Estimated EQ-5D mean utility values by MMD frequency for erenumab and placebo, mapped from MSQ. *Abbreviations: CI* confidence interval, *EQ-5D* 5-dimension EuroQol questionnaire, *FRM* fractional response model, *MMD* monthly migraine day, *MSQ* Migraine-Specific Quality of Life Questionnaire, *REML* restricted maximum likelihood. Observed values with 95% CIs are represented by blue, green and red vertical lines for erenumab 140 mg and 70 mg and placebo, respectively. (**a**) Linear mixed effects model with REML, (**b**) FRM (logit), (**c**) FRM (probit), (**d**) Beta regression model

**Fig. 2:**
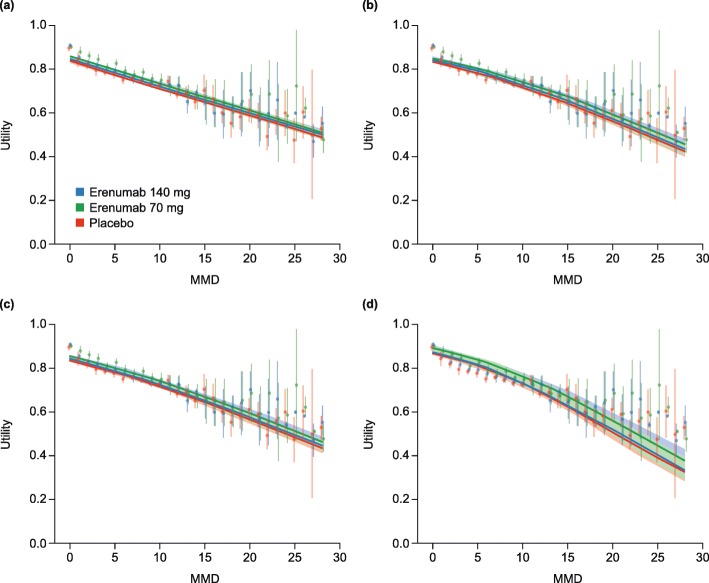
Estimated EQ-5D mean utility values by MMD frequency for erenumab and placebo, mapped from HIT-6. *Abbreviations: CI* confidence interval, *EQ-5D* 5-dimension EuroQol questionnaire, *HIT-6* Headache Impact Test, *MMD* monthly migraine day, *REML* restricted maximum likelihood. Observed values with 95% CIs are represented by blue, green and red vertical lines for erenumab 140 mg and 70 mg and placebo, respectively. (**a**) Linear mixed effects model with REML (**b**) Fractional response model with logit link, (**c**) Fractional response model with probit link and (**d**) beta regression model

The predicted mean utility values by number of MMD are shown in Figs 1and 2 after mapping from MSQ and HIT-6, respectively. Mapped utility values for erenumab patients were consistently higher than for placebo patients with the same number of MMD. All models tested showed similar fittings and fit the observed data well (Figs 1 and 2). Because of the different likelihood functions used for the four regression models proposed in this analysis, the fittings could not be compared via Akaike Information Criterion (AIC) or Bayesian Information Criterion (BIC).

The regression outputs for the utility values after mapping from MSQ and HIT-6 are shown in Tables 3 and 4, respectively. In all models tested, the treatment effect of erenumab 140 mg compared with placebo was significantly higher when mapped from HIT-6 (Table 4). Treatment with erenumab 140 mg compared with placebo was also significantly higher in all models tested when mapped from MSQ (Table 3). Treatment with erenumab 70 mg compared with placebo was not significant when mapped from MSQ or HIT-6 (Tables 3 and 4). Baseline MMD was significant in all models tested apart from the linear model when mapped from HIT-6 (Table 3). RMSEs were similar in the four models assessed (0.115, 0.114, 0.114 and 0.114 for the mixed linear effects model with REML, fractional response model with logit link, fractional response model with probit link and beta regression model, respectively, when mapped from MSQ (Table 3). MAE for the four models tested were also similar when mapped from MSQ (0.086, 0.085, 0.085 and 0.085) and HIT-6 and (0.087, 0.088, 0.088 and 0.089) for the mixed linear effects model with REML, fractional response model with logit link, fractional response model with probit link and beta regression model, respectively.

**Table 3:** Regression outputs mapped from MSQ (*N* = 10,977)

Utility values mapped from MSQ	Linear mixed effects model with REML				Fractional response model (logit)				Fractional response model (probit)				Beta regression			
	Coeff	95% CI		*p* value	Coeff	95% CI		*p* value	Coeff	95% CI		*p* value	*Coeff*	95% CI		*p* value
Erenumab 70 mg (vs placebo)	0.006	−0.004	0.016	0.212	0.0331	−0.015	0.814	0.178	0.020	− 0.089	0.048	0.176	0.023	−0.023	0.068	0.335
Erenumab 140 mg (vs placebo)	0.015	0.004	0.026	0.007	0.0744	0.023	0.125	0.004	0.044	0.0139	0.074	0.004	0.061	0.014	0.109	0.011
Baseline MMD	0.0017	−0.003	−0.001	< 0.001	0.0001	− 0.005	0.005	0.970	−0.0004	−0.004	0.003	0.806	−0.002	−0.007	0.004	0.557
MMD	−0.015	−0.016	−0.015	< 0.001	−0.077	−0.082	− 0.072	< 0.001	− 0.047	− 0.049	− 0.044	< 0.001	− 0.074	− 0.079	− 0.069	< 0.001
Visit																
Week 4	0.037	0.033	0.042	< 0.001	0.167	0.141	0.193	< 0.001	0.101	0.086	0.117	< 0.001	0.160	0.133	0.187	< 0.001
Week 8	0.038	0.033	0.426	< 0.001	0.173	0.144	0.202	< 0.001	0.104	0.087	0.122	< 0.001	0.164	0.134	0.194	< 0.001
Week 12	0.038	0.033	0.042	< 0.001	0.176	0.145	0.206	< 0.001	0.106	0.087	0.124	< 0.001	0.165	0.134	0.197	< 0.001
Week 16	0.032	0.026	0.038	< 0.001	0.194	0.153	0.235	< 0.001	0.115	0.091	0.139	< 0.001	0.178	0.138	0.217	< 0.001
Week 20	0.030	0.024	0.036	< 0.001	0.187	0.148	0.227	< 0.001	0.111	0.087	0.134	< 0.001	0.170	0.133	0.209	< 0.001
Week 24	0.028	0.022	0.035	< 0.001	0.185	0.144	0.227	< 0.001	0.109	0.085	0.134	< 0.001	0.169	0.129	0.209	< 0.001
Age	0.0004	0.0002	0.0008	< 0.001	0.002	−7.11e^−06^	0.004	0.051	0.001	0.000	0.002	0.044	0.002	−0.001	0.003	0.074
Female	−0.015	−0.027	− 0.003	0.011	−0.082	−0.140	−0.024	0.006	−0.048	−0.082	−0.014	0.006	−0.077	−0.130	−0.024	0.004
Race (vs white)																
Black	−0.013	−0.030	0.004	0.140	−0.054	−0.139	0.032	0.217	− 0.033	−0.083	0.018	0.205	−0.050	−0.130	0.030	0.221
Other	−0.022	−0.049	0.005	0.104	−0.129	−0.282	0.024	0.099	−0.077	−0.168	0.135	0.095	−0.109	−0.257	0.039	0.148
RMSE	0.115				0.114				0.114				0.114			
MAE	0.086				0.085				0.085				0.085			

*Abbreviations: CI* confidence interval, *Coeff* coefficient, *MAE* mean absolute error, *MMD* monthly migraine day, *MSQ* Migraine-Specific Quality of Life Questionnaire, *REML* restricted maximum likelihood, *RMSE* root mean squared error

**Table 4:** Regression outputs mapped from HIT-6 (*N* = 10,971)

Utility values mapped from HIT-6	Linear mixed effects model with REML				Fractional response model (logit)				Fractional response model (probit)				Beta regression			
	Coeff	95% CI		*p* value	Coeff	95% CI		*p* value	Coeff	95% CI		*p* value	Coeff	95% CI		*p* value
Erenumab 70 mg (vs placebo)	0.007	−0.002	0.016	0.151	0.037	−0.013	0.870	0.148	0.022	−0.008	0.052	0.143	0.032	−0.058	0.123	0.484
Erenumab 140 mg (vs placebo)	0.020	0.009	0.030	< 0.001	0.112	0.053	0.171	< 0.001	0.067	0.032	0.101	0.0002	0.202	0.076	0.328	0.002
Baseline MMD	0.0002	−0.001	0.001	0.618	0.010	0.004	0.016	0.001	0.005	0.002	0.009	0.003	0.045	0.034	0.057	< 0.001
MMD	−0.012	−0.013	− 0.012	< 0.001	−0.070	−0.075	−0.065	< 0.001	−0.042	−0.045	−0.039	< 0.001	−0.096	−0.107	−0.086	< 0.001
Visit																
Week 4	0.032	0.028	0.037	< 0.001	0.149	0.125	0.174	< 0.001	0.089	0.074	0.104	< 0.001	0.149	0.086	0.211	< 0.001
Week 8	0.040	0.036	0.045	< 0.001	0.195	0.166	0.223	< 0.001	0.116	0.099	0.133	< 0.001	0.244	0.173	0.316	< 0.001
Week 12	0.043	0.038	0.048	< 0.001	0.210	0.180	0.240	< 0.001	0.124	0.107	0.142	< 0.001	0.249	0.178	0.321	< 0.001
Week 16	0.03	0.046	0.059	< 0.001	0.312	0.266	0.357	< 0.001	0.181	0.155	0.208	< 0.001	0.512	0.402	0.621	< 0.001
Week 20	0.050	0.044	0.057	< 0.001	0.306	0.260	0.353	< 0.001	0.178	0.151	0.205	< 0.001	0.529	0.411	0.647	< 0.001
Week 24	0.050	0.044	0.056	< 0.001	0.309	0.261	0.356	< 0.001	0.180	0.152	0.207	< 0.001	0.472	0.369	0.574	< 0.001
Age	0.0007	0.0003	0.001	< 0.001	0.004	0.002	0.006	< 0.001	0.002	0.001	0.004	< 0.001	0.006	0.001	0.010	0.010
Female	−0.023	0.034	−0.012	< 0.001	−0.135	−0.201	−0.069	< 0.001	−0.080	−0.118	−0.041	< 0.001	−0.128	−0.252	− 0.004	0.042
Race (vs white)																
Black	−0.014	−0.030	0.003	0.101	−0.061	−0.155	0.033	0.203	−0.036	−0.091	0.019	0.198	0.021	−0.149	0.191	0.810
Other	−0.003	−0.028	0.227	0.829	−0.016	−0.178	0.145	0.845	−0.008	−0.865	0.086	0.865	0.244	−0.133	0.622	0.205
RMSE	0.112				0.113				0.113				0.118			
MAE	0.087				0.088				0.088				0.089			

*Abbreviations: CI* confidence interval, *Coeff* coefficient, *HIT-6* Headache Impact Test, *MAE* mean absolute error, *MMD* monthly migraine day, *MSQ* Migraine-Specific Quality of Life Questionnaire, *REML* restricted maximum likelihood, *RMSE* root mean squared error

### Multiple imputation analyses

The analyses based on the multiple imputed data sets were substantially similar to the complete case analyses for MSQ and HIT-6 (see Online Resource: Additional file 3: Table S2 and Additional file 4: Table S3). In the regression analyses, there were 10,977 and 10,971 complete case records, when mapped from MSQ and HIT-6, respectively. There were similar proportions of missing observations in the multiple imputed data sets (200 [1.7%] and 205 [1.8%] when mapped from MSQ and HIT-6, respectively) compared with the complete case analysis (200 [7.5%] and 205 [7.5%] when mapped from MSQ and HIT-6, respectively).

Not all patients completed all the scheduled visits: 25 (3.7%), 88 (9.2%) and 30 (5.2%) patients did not complete the planned assessments in the phase 2 study (which was planned for four visits), STRIVE (planned for seven visits) and ARISE (planned for four visits), respectively.

### Discussion

Our analysis describes the assessment of longitudinal approaches in modelling utility values that go beyond simple linear models. In all cases, utility values decreased as the number of MMD increased, and these associations were non-linear with potential ceiling effects. The improvement in average utility values over time in the placebo groups is consistent with the placebo effect on mean MMD frequency observed in the clinical studies [23, 24, 28]. Consistently, mapped utility values for patients treated with erenumab 70 mg and 140 mg were higher than those for participants treated with placebo with the same number of MMD, although only the 140 mg dose of erenumab was significant. This finding is consistent with utility values applied in a previous economic model for onabotulinumtoxin A, which assumed an additional treatment effect of active treatment compared with placebo [46]. This additional treatment effect is most likely driven by improvements in migraine duration and severity, that may not be fully captured by the primary clinical endpoint, MMD.

All models tested showed very similar fittings, although the beta regression model may be considered as the optimal candidate for longitudinal and bounded data, because the beta regression model has the flexibility of a beta distribution model and has previously been used to model quality-adjusted life-years in health economic studies [41, 47]. To determine the generalizability of this model, it would be necessary to examine mapped utility values from other study data.

Some limitations of the analysis have to be acknowledged. Firstly, the HIT-6 and MSQ scores were captured only monthly in the three clinical studies. It may be beneficial to capture HRQoL data more frequently to accurately capture patients’ experiences within the 1-month time periods [34, 48]. This is particularly relevant, because time and other factors can influence how individuals with migraine can report their HRQoL [34]. Secondly, the use of likelihood-based statistics such as AIC/BIC could not be used to compare models with different likelihood functions. The analysis is further limited by the duration of the erenumab clinical studies: longer studies may be able allow more robust models to be fitted. Finally, because there were very similar RMSEs between the models, it was important to assess the non-linear associations between utilities and MMD. In future studies, it would be useful to assess any longer-term time trends, introducing a specific fixed-effect covariate and assessing the potential interaction between treatment and MMD. Exploration of such models as response mappings to predict the levels of utilities would be of interest. Future studies that examine mapping from a measure such as the Migraine Physical Function Impact Diary, which is a daily, migraine-specific measurement of patient-reported outcomes, would also be worth considering [48].

The analysis described here has applications for economic evaluations. Cost-utility analysis is widely recognized as a useful approach for measuring and comparing the efficiency of different health interventions [49]. Furthermore, longitudinal approaches for modelling utilities can be appropriate when considering economic evaluations because they can capture changes in health utility over time. In using utility values that are useful for decision-making bodies, the robust findings of this analysis, consistent across the models fitted, demonstrate the value of this data for health economic evaluations for migraine prevention and treatment.

## Conclusions

Our analysis showed that all models fitted the observed data well. Mapped utility values for patients receiving erenumab were higher than those for patients with the same number of MMD receiving placebo, indicating that treating migraine may have benefit beyond simply reducing the number of migraines a patient experiences and may translate into improvements in HRQoL. Linking patient utility values to the number of MMD allows utility estimates for different levels of MMD to be predicted, for use in economic evaluations of preventive therapies. More broadly, the analysis demonstrates the application of different models for fitting utilities from study data.

## Supplementary information

**Additional file 1: Figure S1.** Distribution of mapped utilities for MSQ and HIT-6. *Abbreviations: EQ-5D* 5-dimension EuroQol questionnaire, *HIT-6* Headache Impact Test, *MSQ* Migraine-Specific Quality of life Questionnaire.

**Additional file 2: Table S1.** Mapping algorithm to estimate EQ-5D utility values from HIT-6 and MSQ [34]

**Additional file 3: Table S2.** Multiple imputation outputs for MSQ.

**Additional file 4: Table S3.** Multiple imputation outputs for HIT-6.

## Data Availability

Data Availability Statement. Qualified researchers may request data from Amgen clinical studies. Complete details are available at the following: http://www.amgen.com/datasharing

## Abbreviations

AIC: Akaike Information Criterion
ARISE: A phase 3, Randomized, double-blind, placebo-controlled Study to Evaluate the efficacy and safety of AMG 334 in migraine prevention
BIC: Bayesian Information Criterion
CI: Confidence interval
CM: Chronic migraine
Coeff: Coefficient
EM: Episodic migraine
EQ-5D: 5 dimension EuroQol questionnaire
FCS: Fully conditional specification
FRM: Fractional response model
GLM: Generalized linear model
HD: Headache day
HIT-6: Headache Impact Test
HRQoL: Health-related quality of life
HUI: Health Utilities Index
MAE: Mean absolute error
MICE: Multivariate imputation by chained eqs.
MMD: Monthly migraine day
MSQ: Migraine-Specific Quality of Life Questionnaire
NCT: National clinical trial
REML: Restricted maximum likelihood
RMSE: Root mean squared error
SD: Standard deviation
STRIVE: Study to evaluate the efficacy and safety of erenumab in migraine prevention
