# Supplementary material for: Longitudinal assessment of utilities in patients with migraine: an analysis of erenumab randomized controlled trials
Source: Health Qual Life Outcomes. 2019 Nov 12;17:171. doi: 10.1186/s12955-019-1242-6 (PMC6852901; doi:10.1186/s12955-019-1242-6)
Supplement: Supplementary file 2 — Additional file 2: Table S1. Mapping algorithm to estimate EQ-5D utility values from HIT-6 and MSQ [34] [file 12955_2019_1242_MOESM2_ESM.docx]

**Supplementary Table 1** Mapping algorithm^*^ to estimate EQ-5D utility values from HIT-6 (a) and MSQ (b) [34]

(a)

|  | Episodic migraine | Chronic migraine |
| --- | --- | --- |
| N | 5961 | 373 |
| Intercept | 1.6055* | 2.5525* |
| HIT-6 | −0.01483* | −0.0313* |
| Adjusted R^2^ | 0.08 | 0.19 |
| RMSE | 0.32 | 0.33 |

(b)

|  | Episodic migraine | Chronic migraine |
| --- | --- | --- |
| N | 5770 | 338 |
| Intercept | 0.2858^#^ | −0.0492 |
| MSQ-RP | 0.0029^#^ | 0.0065^#^ |
| MSQ-RR | 0.0001 | 0.0013 |
| MSQ-EF | 0.0027^#^ | 0.0011 |
| Adjusted R^2^ | 0.14 | 0.30 |
| RMSE | 0.32 | 0.32 |

*Abbreviations: EF* emotional function, *EQ-5D* 5-dimension EuroQol questionnaire, *HIT-6* Headache Impact Test, *MSQ* Migraine-Specific Quality of life Questionnaire, *RMSE* root mean squared error, *RP* role prevention, *RR* role restriction
^*^The mapping algorithm has been previously validated and published by Gillard et al. [30]
^#^*p* ≤ 0.05
(a) EQ-5D questionnaire = Intercept + HIT-6 + error
(b) EQ-5D questionnaire = Intercept + MSQ-RP + MSQ-RR + MSQ-EF + error
