# Supplementary material for: Longitudinal assessment of utilities in patients with migraine: an analysis of erenumab randomized controlled trials
Source: Health Qual Life Outcomes. 2019 Nov 12;17:171. doi: 10.1186/s12955-019-1242-6 (PMC6852901; doi:10.1186/s12955-019-1242-6)
Supplement: Supplementary file 3 — Additional file 3: Table S2. Multiple imputation outputs for MSQ. [file 12955_2019_1242_MOESM3_ESM.docx]

**Supplementary Table 2** Multiple imputation outputs for MSQ (*N* = 11 647).

| Multiple imputation estimates for MSQ | Linear mixed effects model with REML | | | | Fractional response model (logit) | | | | Fractional response model (probit) | | | | Beta regression | | | |
| --- | --- | --- | --- | --- | --- | --- | --- | --- | --- | --- | --- | --- | --- | --- | --- | --- |
|  | Coeff | 95% CI | | *p* value | Coeff | 95% CI | | *p* value | Coeff | 95% CI | | *p* value | Coeff | 95% CI | | *p* value |
| Erenumab 70 mg (vs placebo) | 0.060 | −0.004 | 0.016 | 0.225 | 0.029 | −0.020 | 0.077 | 0.246 | 0.017 | −0.012 | 0.046 | 0.243 | 0.020 | −0.026 | 0.066 | 0.402 |
| Erenumab 140 mg (vs placebo) | 0.016 | 0.004 | 0.027 | 0.004 | 0.079 | 0.028 | 0.130 | 0.002 | 0.047 | 0.017 | 0.077 | 0.002 | 0.066 | 0.018 | 0.113 | 0.007 |
| Baseline MMD | −0.002 | −0.003 | −0.001 | < 0.001 | < 0.001 | −0.005 | 0.006 | 0.815 | < 0.001 | −0.003 | 0.003 | 0.965 | −0.001 | −0.006 | 0.004 | 0.700 |
| MMD | −0.015 | −0.016 | −0.015 | < 0.001 | −0.078 | −0.083 | −0.073 | < 0.001 | −0.047 | −0.050 | −0.044 | < 0.001 | −0.075 | −0.080 | −0.070 | < 0.001 |
| Visit |  | | | |  | | | |  | | | |  |  |  |  |
| Week 4 | 0.037 | 0.032 | 0.041 | < 0.001 | 0.159 | 0.133 | 0.184 | < 0.001 | 0.096 | 0.081 | 0.112 | < 0.001 | 0.152 | 0.126 | 0.179 | < 0.001 |
| Week 8 | 0.037 | 0.032 | 0.042 | < 0.001 | 0.162 | 0.133 | 0.190 | < 0.001 | 0.098 | 0.081 | 0.115 | < 0.001 | 0.153 | 0.124 | 0.182 | < 0.001 |
| Week 12 | 0.037 | 0.032 | 0.041 | < 0.001 | 0.160 | 0.130 | 0.189 | < 0.001 | 0.096 | 0.078 | 0.114 | < 0.001 | 0.151 | 0.120 | 0.181 | < 0.001 |
| Week 16 | 0.032 | 0.026 | 0.038 | < 0.001 | 0.181 | 0.141 | 0.220 | < 0.001 | 0.107 | 0.084 | 0.131 | < 0.001 | 0.167 | 0.128 | 0.205 | < 0.001 |
| Week 20 | 0.030 | 0.024 | 0.036 | < 0.001 | 0.169 | 0.129 | 0.208 | < 0.001 | 0.100 | 0.076 | 0.124 | < 0.001 | 0.155 | 0.116 | 0.194 | < 0.001 |
| Week 24 | 0.027 | 0.021 | 0.033 | < 0.001 | 0.155 | 0.115 | 0.196 | < 0.001 | 0.092 | 0.068 | 0.116 | < 0.001 | 0.144 | 0.104 | 0.183 | < 0.001 |
| Age | < 0.001 | < 0.001 | < 0.001 | 0.023 | 0.002 | < 0.001 | 0.004 | 0.020 | 0.001 | < 0.001 | 0.002 | 0.171 | 0.002 | < 0.001 | 0.004 | 0.034 |
| Female | −0.015 | −0.026 | −0.003 | 0.013 | −0.083 | −0.141 | −0.024 | 0.005 | −0.049 | −0.083 | −0.014 | 0.006 | −0.077 | −0.131 | −0.024 | 0.005 |
| Race (vs white) |  |  |  |  |  |  |  |  |  |  |  |  |  |  |  |  |
| Black | −0.014 | −0.031 | 0.003 | 0.110 | −0.069 | −0.157 | 0.019 | 0.122 | −0.042 | −0.094 | 0.010 | 0.116 | −0.065 | −0.148 | 0.018 | 0.123 |
| Other | −0.025 | −0.051 | 0.002 | 0.070 | −0.133 | −0.279 | 0.011 | 0.071 | −0.081 | −0.167 | 0.006 | 0.066 | −0.116 | −0.255 | 0.024 | 0.104 |

Abbreviations: *CI* Confidence interval, *Coeff* Coefficient, *MMD* Monthly migraine day, *MSQ* Migraine-Specific Quality of Life Questionnaire, *REML* Restricted maximum likelihood
The proportions of missing observations were as follows: mapped MSQ, 200 (7.5%); treatment, 0 (0%); baseline MMD, 0 (0%); MMD, 85 (6.5%); age, 0 (0%); gender, 0 (0%); race, 0 (0%)
