# Supplementary material for: Longitudinal assessment of utilities in patients with migraine: an analysis of erenumab randomized controlled trials
Source: Health Qual Life Outcomes. 2019 Nov 12;17:171. doi: 10.1186/s12955-019-1242-6 (PMC6852901; doi:10.1186/s12955-019-1242-6)
Supplement: Supplementary file 4 — Additional file 4: Table S3. Multiple imputation outputs for HIT-6. [file 12955_2019_1242_MOESM4_ESM.docx]

**Supplementary Table 3** Multiple imputation outputs for HIT-6 (*N* = 11 647).

| Multiple imputation estimates for HIT-6 | Linear mixed effects model with REML | | | | Fractional response model (logit) | | | | Fractional response model (probit) | | | | Beta regression | | | |
| --- | --- | --- | --- | --- | --- | --- | --- | --- | --- | --- | --- | --- | --- | --- | --- | --- |
|  | Coeff | 95% CI | | *p* value | Coeff | 95% CI | | *p* value | Coeff | 95% CI | | *p* value | Coeff | 95% CI | | *p* value |
| Erenumab 70 mg (vs placebo) | 0.006 | −0.003 | 0.015 | 0.179 | 0.283 | −0.020 | 0.077 | 0.248 | 0.017 | −0.012 | 0.045 | 0.247 | 0.032 | −0.020 | 0.084 | 0.228 |
| Erenumab 140 mg (vs placebo) | 0.175 | 0.007 | 0.028 | < 0.001 | 0.096 | 0.040 | 0.152 | < 0.001 | 0.057 | 0.024 | 0.090 | < 0.001 | 0.101 | 0.041 | 0.161 | 0.001 |
| Baseline MMD | < 0.001 | −0.001 | < 0.001 | 0.285 | 0.005 | −0.001 | 0.011 | 0.067 | 0.003 | −0.001 | 0.006 | 0.113 | 0.007 | 0.001 | 0.013 | 0.019 |
| MMD | −0.012 | −0.124 | −0.011 | < 0.001 | −0.065 | −0.070 | −0.061 | < 0.001 | −0.039 | −0.042 | −0.036 | < 0.001 | −0.064 | −0.069 | −0.059 | < 0.001 |
| Visit |  | | | |  | | | |  | | | |  |  |  |  |
| Week 4 | 0.032 | 0.027 | 0.036 | < 0.001 | 0.143 | 0.119 | 0.168 | < 0.001 | 0.086 | 0.711 | 0.101 | < 0.001 | 0.156 | 0.129 | 0.184 | < 0.001 |
| Week 8 | 0.039 | 0.034 | 0.043 | < 0.001 | 0.181 | 0.153 | 0.210 | < 0.001 | 0.109 | 0.092 | 0.126 | < 0.001 | 0.197 | 0.167 | 0.228 | < 0.001 |
| Week 12 | 0.041 | 0.037 | 0.046 | < 0.001 | 0.197 | −0.168 | 0.227 | < 0.001 | 0.117 | 0.100 | 0.135 | < 0.001 | 0.225 | 0.193 | 0.257 | < 0.001 |
| Week 16 | 0.048 | 0.041 | 0.054 | < 0.001 | 0.277 | 0.232 | 0.321 | < 0.001 | 0.162 | 0.136 | 0.188 | < 0.001 | 0.304 | 0.257 | 0.350 | < 0.001 |
| Week 20 | 0.046 | 0.040 | 0.052 | < 0.001 | 0.269 | 0.224 | 0.315 | < 0.001 | 0.158 | 0.131 | 0.184 | < 0.001 | 0.305 | 0.259 | 0.350 | < 0.001 |
| Week 24 | 0.045 | 0.039 | 0.052 | < 0.001 | 0.267 | 0.220 | 0.314 | < 0.001 | 0.156 | 0.129 | 0.184 | < 0.001 | 0.294 | 0.245 | 0.342 | < 0.001 |
| Age | < 0.001 | < 0.001 | 0.001 | < 0.001 | 0.004 | 0.002 | 0.006 | < 0.001 | 0.002 | 0.001 | 0.004 | < 0.001 | 0.004 | 0.002 | 0.006 | < 0.001 |
| Female | −0.022 | −0.032 | −0.011 | < 0.001 | −0.128 | −0.191 | −0.065 | < 0.001 | −0.076 | −0.112 | −0.039 | < 0.001 | −0.129 | −0.193 | −0.066 | < 0.001 |
| Race (vs white) |  |  |  |  |  |  |  |  |  |  |  |  |  |  |  |  |
| Black | −0.015 | −0.032 | < 0.001 | 0.064 | −0.079 | −0.171 | 0.130 | 0.092 | −0.471 | −0.101 | 0.007 | 0.089 | −0.067 | −0.160 | 0.256 | 0.156 |
| Other | −0.007 | −0.032 | 0.018 | 0.562 | −0.049 | −0.193 | 0.940 | 0.500 | −0.295 | −0.113 | 0.054 | 0.488 | −0.027 | −0.206 | 0.152 | 0.769 |

*Abbreviations: CI* Confidence interval, *Coeff* Coefficient, *HIT-6* Headache Impact Test, *MMD* Monthly migraine day
The proportions of missing observations were as follows: mapped HIT-6, 205 (7.5%); treatment, 0 (0%); baseline MMD, 0 (0%); MMD, 85 (6.5%); age, 0 (0%); gender, 0 (0%); race, 0 (0%)
